# 3D Reconstruction of Coronary Artery Vascular Smooth Muscle Cells

**DOI:** 10.1371/journal.pone.0147272

**Published:** 2016-02-16

**Authors:** Tong Luo, Huan Chen, Ghassan S. Kassab

**Affiliations:** 1 Biomedical Engineering, IUPUI, Indianapolis, IN, United States of America; 2 Surgery, IUPUI, Indianapolis, IN, United States of America; 3 Cellular and Integrative Physiology, IUPUI, Indianapolis, IN, United States of America; Pennsylvania State Hershey College of Medicine, UNITED STATES

## Abstract

**Aims:**

The 3D geometry of individual vascular smooth muscle cells (VSMCs), which are essential for understanding the mechanical function of blood vessels, are currently not available. This paper introduces a new 3D segmentation algorithm to determine VSMC morphology and orientation.

**Methods and Results:**

A total of 112 VSMCs from six porcine coronary arteries were used in the analysis. A 3D semi-automatic segmentation method was developed to reconstruct individual VSMCs from cell clumps as well as to extract the 3D geometry of VSMCs. A new edge blocking model was introduced to recognize cell boundary while an edge growing was developed for optimal interpolation and edge verification. The proposed methods were designed based on Region of Interest (ROI) selected by user and interactive responses of limited key edges. Enhanced cell boundary features were used to construct the cell’s initial boundary for further edge growing. A unified framework of morphological parameters (dimensions and orientations) was proposed for the 3D volume data. Virtual phantom was designed to validate the tilt angle measurements, while other parameters extracted from 3D segmentations were compared with manual measurements to assess the accuracy of the algorithm. The length, width and thickness of VSMCs were 62.9±14.9μm, 4.6±0.6μm and 6.2±1.8μm (mean±SD). In longitudinal-circumferential plane of blood vessel, VSMCs align off the circumferential direction with two mean angles of -19.4±9.3° and 10.9±4.7°, while an out-of-plane angle (i.e., radial tilt angle) was found to be 8±7.6° with median as 5.7°.

**Conclusions:**

A 3D segmentation algorithm was developed to reconstruct individual VSMCs of blood vessel walls based on optical image stacks. The results were validated by a virtual phantom and manual measurement. The obtained 3D geometries can be utilized in mathematical models and leads a better understanding of vascular mechanical properties and function.

## Introduction

Mechanical stresses induced in the vessel wall are in part influenced by arrangement and shape of individual vascular smooth muscle cells (VSMCs), most of which are believed to be mainly distributed along the circumference of the vascular wall [1–3]. There has been significant effort, based on 2D histology images [4], to determine cellular morphology to predict vascular mechanical response. The mechanical models, however, require accurate geometrical information of cells exposed to the biomechanical microenvironments of vessel wall [5–6]. It is expected that 3D cellular structure can provide more accurate geometries (e.g., 3D dimensions and orientation) for better understanding of vascular biomechanics. Furthermore, realistic 3D cellular morphology of blood vessels are also essential for understanding pathology of blood vessels, given that the major causes of cardiovascular disease (e.g., hypertension and atherosclerosis) typically involve hypertrophy or hyperplasia of VSMCs [7–8]. A 2D view of cells is limited and complex geometric features can be more accurately represented based on 3D reconstruction of cells. The latest developments of microscopy technology enable noninvasive visualization of VSMCs [9] and provides high-quality image stacks for computational analysis of 3D reconstruction of individual cells.

A complete VSMC reconstruction requires 3D cell segmentation and surface coordinates [10]. Generally, the studies of 3D model reconstruction have focused on single cells in clumps. Complex and tenuous structure of multiple cells, however, challenge most image analysis methods. Some new algorithms address special VSMCs clump segmentation [11–12], while most 3D models have been reduced to simple geometries and were not segmented from cell samples [13]. An efficient segmentation algorithm is needed to improve accuracy of 3D reconstruction for a single cell.

There exist two main problems in VSMC segmentations: 1) the general segmentation problem must be solved and 2) the special features of VSMC scanned in longitudinal-circumferential plane must be captured. The major challenge is to distinguish boundaries of touching or overlapped objects [14–17]. The limitation of segmentation algorithms in microscope images has been discussed in the literature [17–18], and there are inter-cell and intra-cell intensity variances (intersection of grey levels among different objects), such as adjacent cells and background. Generally, weak boundaries typically occur in object neighboring. Specifically, cell clumps containing multiple cells with various shapes, sizes, orientations and positions, make complex structures that are difficult to analyze.

Here, we analyzed the geometry and shape of VSMCs by introducing a new edge blocking model for cell clump segmentation algorithm. The cell edge was decomposed into several parts and different strategies were tested. Edge stability and edge growing were proposed to address the edge gap problem. A virtual phantom model was used to validate the measurements of VSMC tilt angles, while the validation of other parameters was made by comparison with manual measurements. The 3D geometries of VSMCs including the width, length, thickness, slenderness ratio, in-plane angle (orientation in longitudinal-circumferential plane) and out-of-plane angle (i.e., radial tilt angles) were extracted based on the 3D reconstruction.

## Methods

### VSMC Image Data

**Fig 1:**
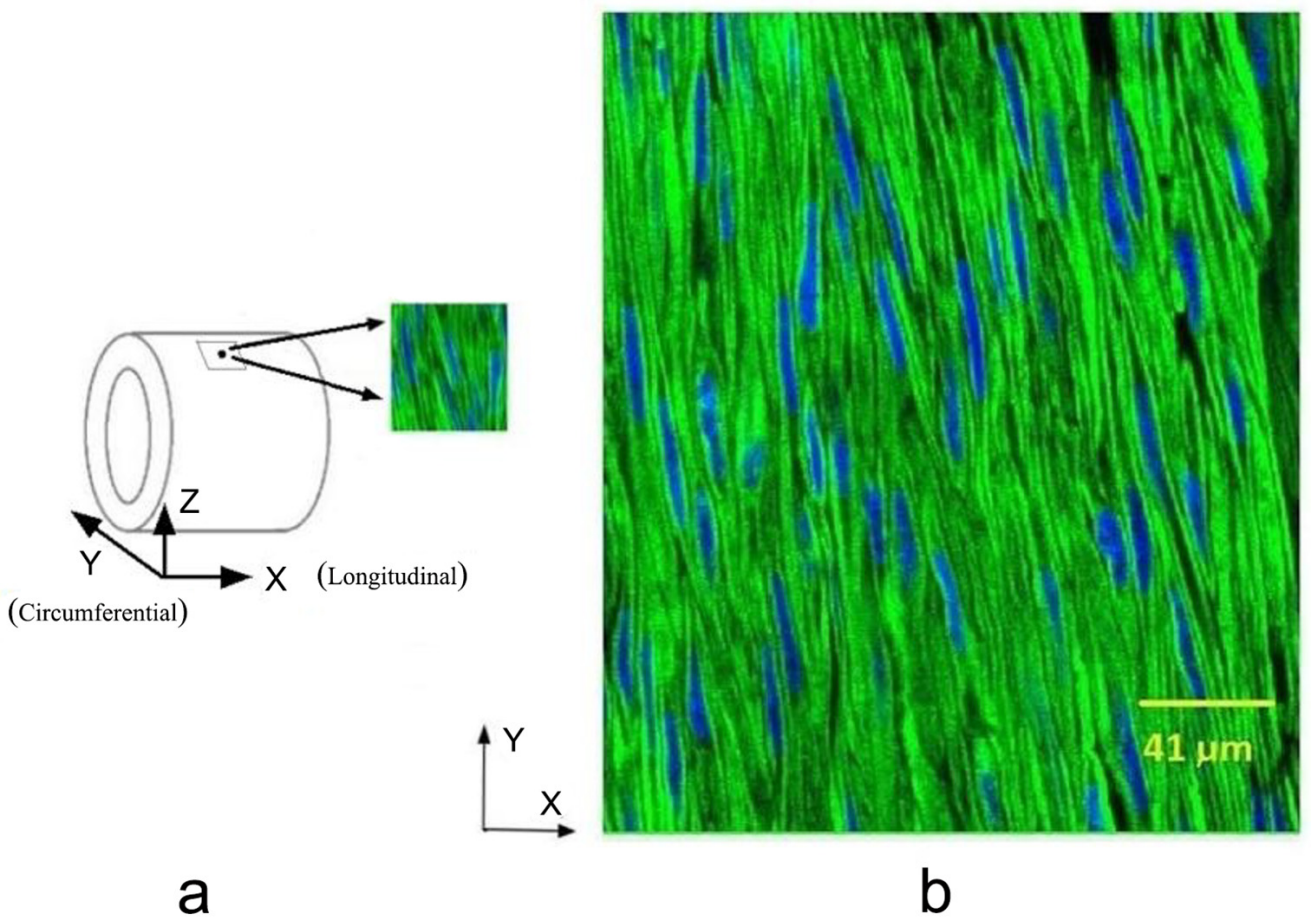
**a)** Vessel were imaged (black dot region) on longitudinal-circumferential plane. X, Y, and Z indicate the longitudinal direction, circumferential directions and radial directions of blood vessel, respectively; **b)** Image with a resolution of 512 × 512 pixels. Blue indicates the nucleus, and green indicates F-actin, i.e., the cell.

Images from six healthy pig (body weight in the range of 80–90 Kg) coronary arteries were analyzed in the present study. The hearts were harvested immediately after sacrifice at a local slaughterhouse (Archers Meats, Greenwood, IN; where our group was located when the measurements were performed). Details have been provided in a previous study [19]. The hearts were then transported to the laboratory in 4°C physiological solution (0.9% NaCl) and the coronary artery segments were dissected carefully from the hearts and immersion-fixed in 4% methanol-free paraformaldehyde solution. 5x5 mm^2^ cross-sections were then sectioned from the segments and the circumferential direction was marked (Fig 1a). The adventitia was peeled off from the sections to facilitate the staining and imaging, and samples were then labeled by two fluorescent probes: phalloidin (excitation/emission wavelength: 495/518 nm) and DAPI (excitation/emission wavelength: 358/461 nm) to capture the F-actin and nucleus of VSMCs (Fig 1b). After staining, the sections were wet mounted on microscope slides using a glycerin-water mixture. The samples were scanned by an FV1000-MPE confocal microscope equipped with multiple laser options and a 60x 1.1NA water immersion objective. Z-stack scans were performed with a 0.25 μm step size, and a scanning spot (i.e., imaging data set) covered a volume of 211x211x10 μm^3^.

### User Input

**Fig 2:**
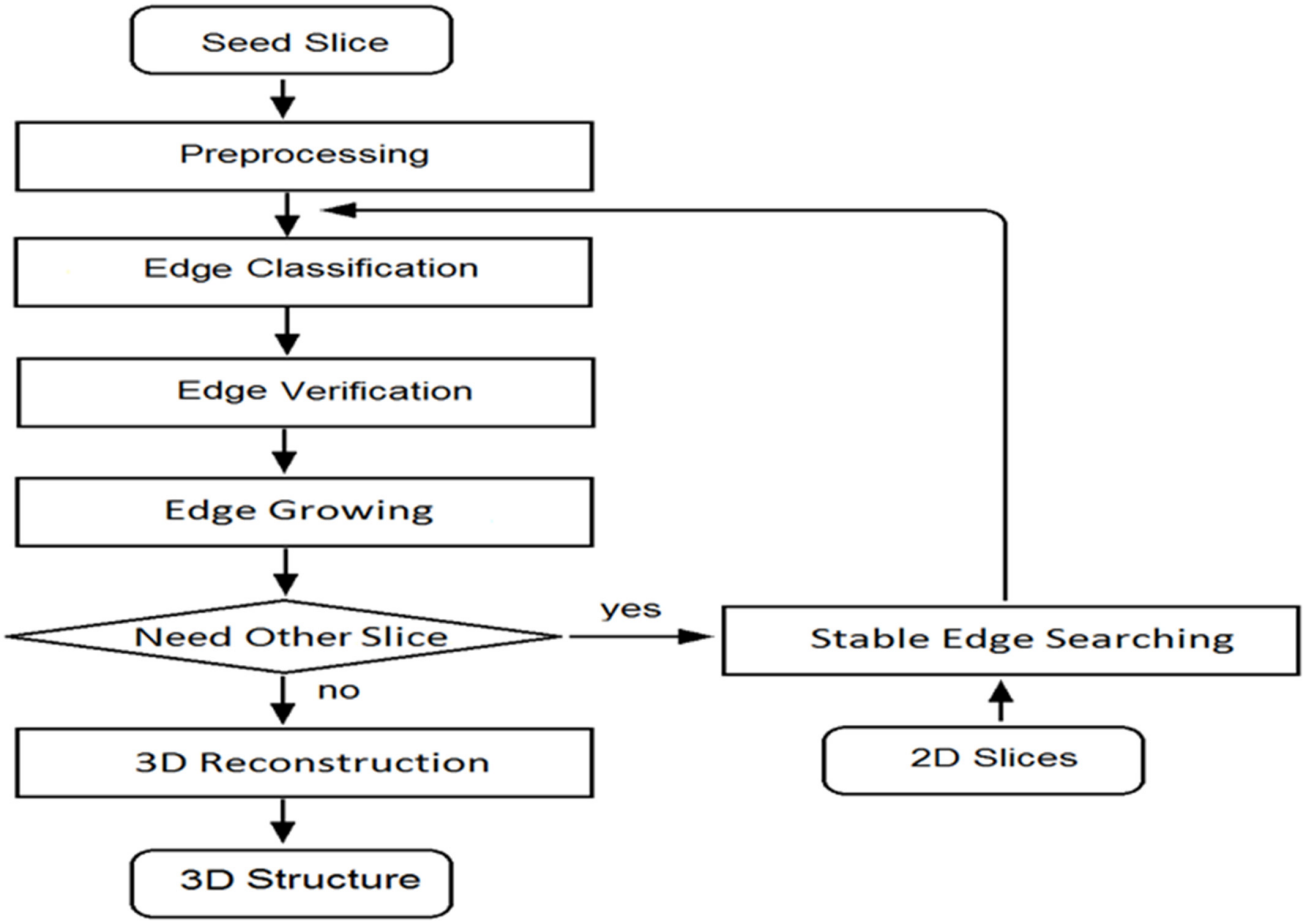
Flow chart of the image processing algorithm.

An ROI on initial slice was drawn by a user in the semi-automation segmentation algorithm. Specific nuclei were selected to be the start points, and edges in ROI were processed and treated as initial edges. A flow chart for image processing is shown in Fig 2 where the primary procedures are introduced in the paragraphs below and the algorithm technical details are presented in S1 Fig. In sequential slices, edges were detected by a newly developed edge blocking method. The edge blocking method may lead to false results under some circumstances, however, such as when some edges overlap with others. Hence, user input was needed to select initial slice or key edges. Slice numbers were selected based on average nucleus thickness. Generally, 13–15 slices were used for a nucleus. Usually, the 2D boundary was composed of dozens of points, manual segmentation cannot have the enough high accuracy and all boundary points were used to sketch out boundary. The computerized gap linking method operated directly on edge segments which were points groups, the time complexity of computerized method is roughly *O(N)* on part of boundary points where the edge detection is revised.

### Output Definition

**Fig 3:**
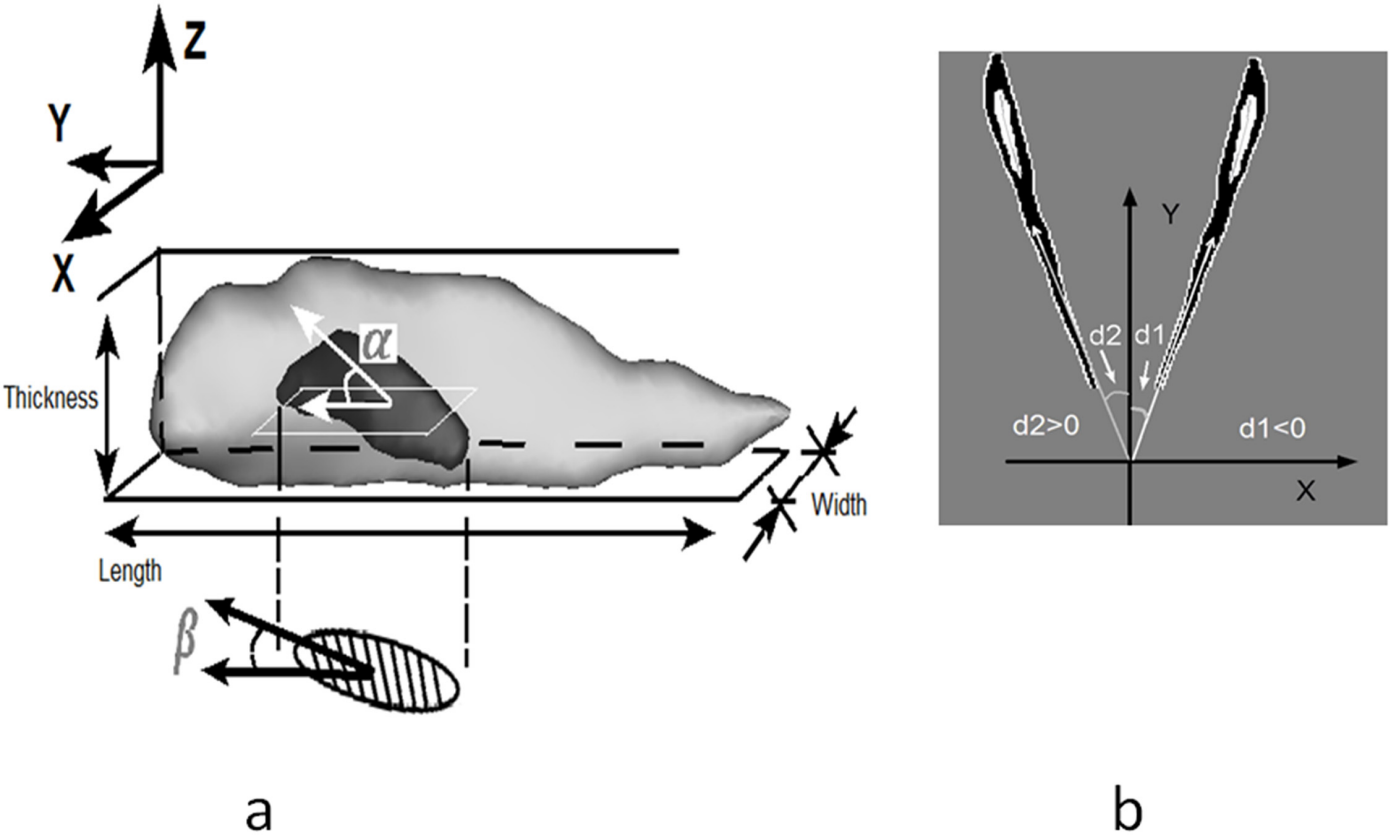
a) 3D surface rendering of nucleus denotes the out-of-plane angle (tilt angle), labeled as **α**, in-plane orientation angle of projected nucleus was denoted as **β**. Shade region below in XY plane is an approximate ellipse for nucleus region. X,Y,Z coordinate axis is consistent with that in Fig 1. The definition of cell length, width and thickness were illustrated. **b)** Two cell with typical in-plane angles were illustrated.

The output of original images was the cell contours in 2D slices and 3D object. The 3D segmentation of entire cell including nucleus and cytoskeletal structures. Based on 2D contours, the 3D object was reconstructed, and smooth surface was obtained for further geometric measurements. The 3D nucleus structure was obtained from nucleus image and used for tilt angle measurements. More details of geometrical parameter extractions are outlined below. The schematic definition of the parameters of VSMC length, width and thickness are illustrated in Fig 3a where the actual 3D shape of the nucleus is presented.

### Extraction of Geometrical Parameters

Out-of-plane and In-plane orientation angles are shown as *α* and *β* in Fig 3a. The projection of a 3D cell on the XY plane (i.e., longitudinal-circumferential plane) approximates an ellipse that consists of a long axis and a short axis by using a MATLAB Function RegionProps. According to spherical coordinate system terminology, in-plane angle is an angle between the long axis and the Y direction (i.e., circumferential direction of the vessel), while out-of-plane angle is an altitude angle out of XY plane. Orientation was also pointed towards the cell blunt pole and there was a deviation from the Y-axis in 2D image coordinates. The in-plane orientation angle was then outputted and ranges from -90 to 90 degree (the circumferential direction is defined as 0) as shown in Fig 3b. The thickness was extracted on height along Z axis; and the length and width were extracted based on 2D projections.

To measure the tilt angle (defined in the range of 0 to 90°), the 3D surface coordinates of cell were used to form 3-by-3 covariance matrix, which measures the position difference between XYZ directions. A Principal component analysis (PCA) was employed to obtain the principal component vector that was equal to the tilt direction. A PCACOV function in MATLAB can be used for the analysis with two options: normalized coordinates with data size or non-normalized coordinates. The accuracy of two options was analyzed in S1 Fig. The tilt angle was extracted based on the nucleus image based on the same approach.

### Edge Blocking Model

**Fig 4:**
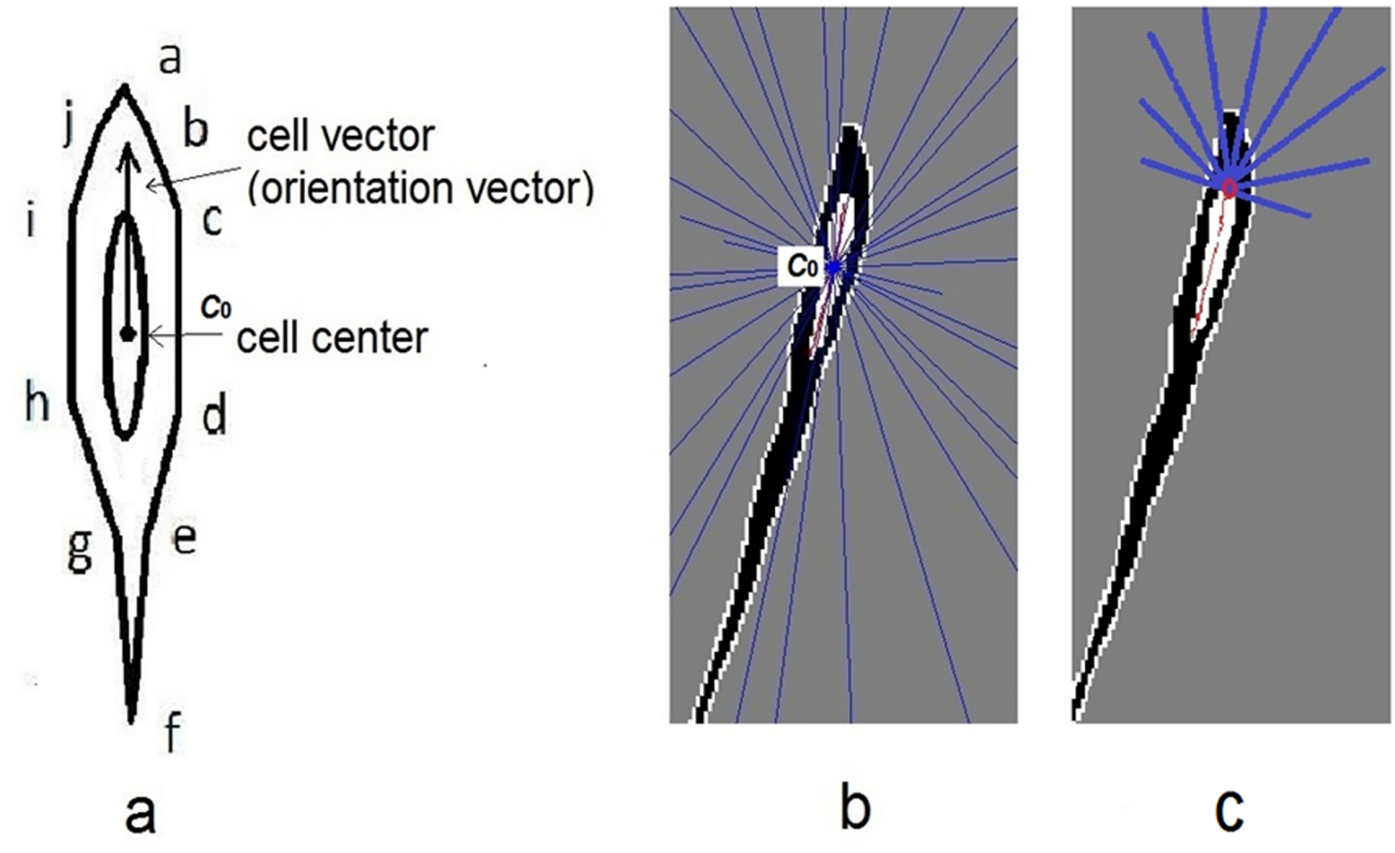
**a)** An idealized shape model of single cell was showed. The right edge was ordered in a-b-c-d-e-f. A trunk edge was b-c-d-e and tail edge was a-b and e-f. The cell center was overlapped by ellipses, it was the nucleus. A vector of the cell direction is indicated by an arrow along the long-axis of the cell. Cell center was denoted as C0. **b)** Blue ray lines emitted from the center, while the nucleus skeleton in red was drawn with two end points. **c)** Extended ray lines were emitted from end point.

An idealized shape model of a single cell is shown in Fig 4a. Longitudinal-circumferential plane was denoted as **Y-X** plane. The nucleus was binarized and overlapped virtually to the cell image (i.e., image of F-actin) at the cell center. Outside the nucleus, the edges or segments symmetrically distributed and the boundary was defined as:

cell boundary={left(right)trunk edge, left(right) tail edge, gap} (1)

The left and right trunk edges were relatively close to the nucleus. Since a cell has varying curvature and width, the inner edges were detected in a cell image in which the intensity was lower in the nucleus region. Nuclei center pixel was defined as the cell center. Each cell was also equalized to a cell vector (orientation vector) from center to one endpoint of long axis. After nucleus region thinning, one skeleton with two end points was extracted by Matlab R2011b Image Processing toolbox function BWMORPH. The function provided specific mathematical morphology operations to the binary image, such as regional thinning and finding endpoint.

An edge blocking model was proposed such that the cell edge segments were intersected by ray lines emitted from center *c*_0_. A cell image with ray lines is illustrated in Fig 4b, in which nucleus skeleton is depicted in red and the blue lines are ray lines. The first intersection points were determined by the cellular edge and ray lines. Edges of adjacent cells and ray lines may also intersect, but their intersections were considered to be blocked by current cell edge. The ray line is defined as a vector, emitted from the geometric center of the nucleus. The two features in ray include scattering angle *θ* and emitting length *l* from the center to intersection point. The angle *θ* will be converted from radian into degree within range of [0 − 360°) as follows:

$$\theta =mod\left[(tan^{-1}\left(\frac{n_{y}}{n_{x}}\right)-tan^{-1}\left(\frac{y-y_{0}}{x-x_{0}}\right)),\;6.28\right]$$ (2)

$$l=lc_{0}\left(1+lc_{1}\text{cos}\left(\theta \right)\right),\;lc_{1}=5;lc_{0}=30$$ (3)

where (*n*_*x*_, *n*_*y*_) denotes cell vector, (*x*, *y*) denotes edge point and (*x*_0_, *y*_0_) denotes cell center. The length unit is pixel and *lc*_1_,*lc*_0_ are determined as the minimal and maximal emitting lengths, respectively. If the ray lines are parallel to edge direction at the tail, new expanded ray lines are emitted from the one end point of skeleton to increase scattering area (Fig 4c).

**Fig 5:**
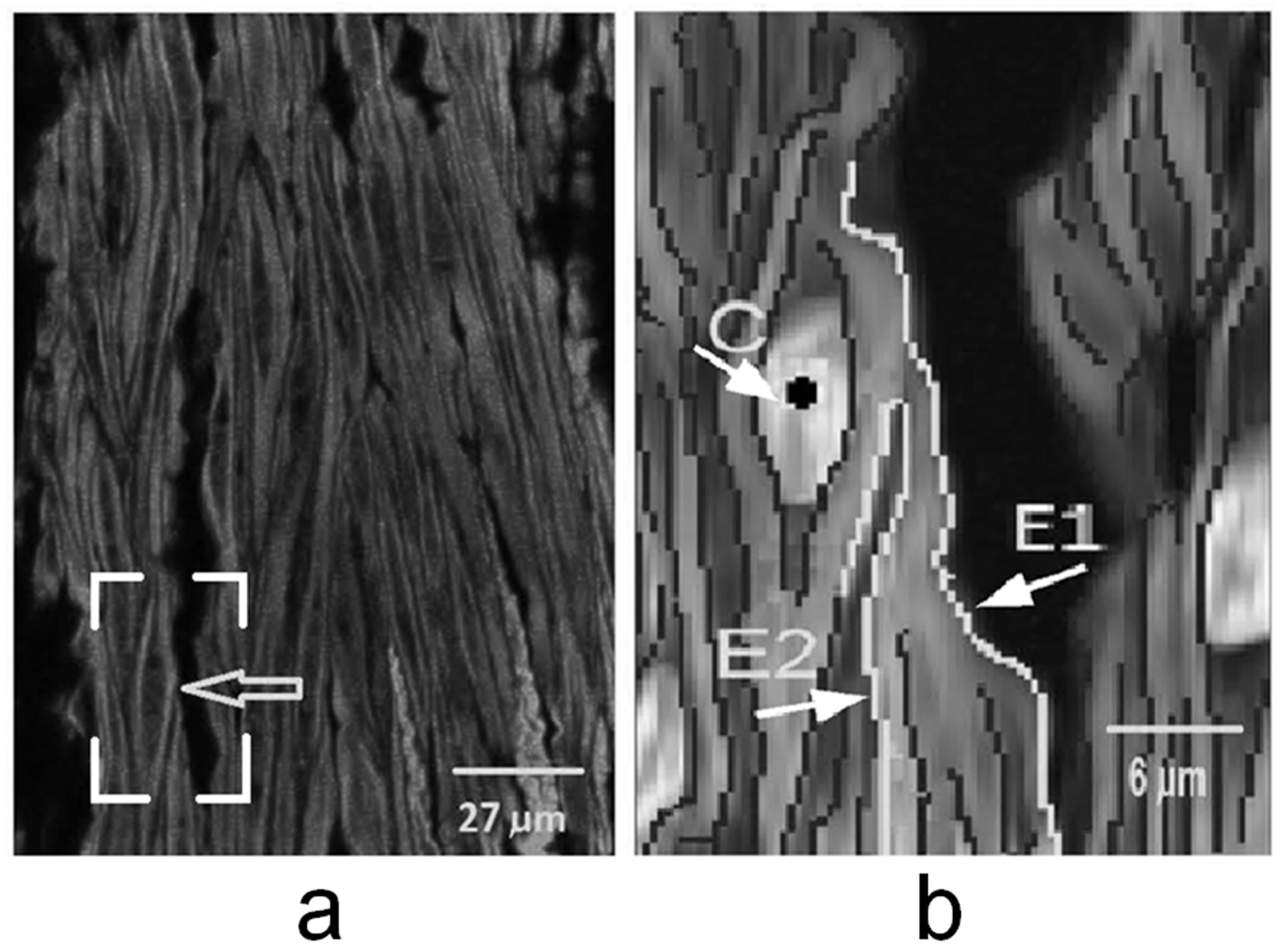
**a)** Original image, arrow indicates the position of Fig 5b. The nuclei were in darker gray and overlaid from separated image. Cell was in gray. **b)** Zoom in region from box frame in Fig 5a, where E1, E2 were edges from two merged cells, and dark dot C was a cell center.

There are two kinds of edge gaps in a cell: 1) Type I is gaps between broken edges of the same cell, and 2) Type II is caused by other cells overlapping or adhering (as shown in Fig 5; Fig 5a is the original image, and Fig 5b is a zoom-in image. E1, E2 are edges of two different cells. Generally, when ray line is emitting from the center point *C*, some parts of E1 and E2 are blocked by cell edge centered at point *C*. Gaps were detected based on the edge blocking model and only unblocked segments were retained and linked later. In this model, block detection was implemented by intersections of rays and edges [20], and it is efficient for segmentation of various shape objects.

### 2D to 3D Segmentation

Cell edges were first processed by using the edge blocking model. The ray lines intersect with three types of edges: inner edge, Type A/B/C edge, or edge of other cells as defined in S1 Fig. The intersection pointes can be ordered according to their distance to the center; i.e., the closest are the first points, and the farthest are the third points. The third points were usually blocked by the first two and treated as noises. The inner edge was connected to the nucleus region and removed by region growing. After the gap type was identified, the corresponding gap can be filled by either Bilateral Spline Interpolation or Laplace verification principal component analysis (PCA).

Anisotropic filtering was done in preprocessing and edge growing was based on different edge category. Edge feature was enhanced by edge verification. The edge in 3D context was searched based on stable edge criteria, where the stable edge was obtained in 2D slices. The segmentations were implemented based on sequential 2D images of cells where adjacent slices provide 3D structural information. The challenge is cell segmentation due to the complex structure, while the segmentation of the nucleus is relatively easy and can be completed by ImageJ process binary toolbox. The 2D boundary of a slice was acquired after edge linking, and then used for 3D segmentation. The 3D surface was rendered by overlapping and filling 2D boundaries of all sequential slices. The MATLAB mathematical morphological operation function of BWMORPH was used to fill gaps and remove noise from binary images. MeshLab was a software tool used to improve the quality of generated 3D mesh and Meshlab global Laplacian smooth was used for initial smoothing and selected face smooth was used to refine the local geometry.

### Validation of Tilt Angle Measurement

The measurement of 3D tilt angle was validated using a virtual phantom model where additional details are provided in S1 Fig. The phantom was a virtual ellipsoid object with accuracy at sub-pixel levels. It is a group of 3D curves that form surface mesh, where the orientation angles are predefined. The surface mesh was converted into sequential 2D images, which were later reconstructed into the 3D object using the proposed 3D algorithm. The tilt angle was extracted based on the reconstructed 3D ellipsoid and compared with the predefined angle.

### Manual Validation of 2D Morphology Parameters

Manual validation is a typical method used in computer segmentation when there is no gold standard [19]. Manual geometry measurements of the same cells on 2D images were performed by a different person (blinded to the automated results) by use of ImageJ to validate the proposed 3D algorithm. A 2D image slice (usually in the middle of a stack) was selected to present 2D geometry of individual cells. The length, width, slenderness ratio (defined as length/width) and in-plane orientation angle of cells were measured directly on 2D images and then compared with the semi-automatic measurements. ImageJ provides measurement toolbox for manual operations. To reduce noise, width was obtained by taking the average of measurements at several positions. The validation was based on statistical results and the mean and variances were compared.

## Results

**Fig 6:**
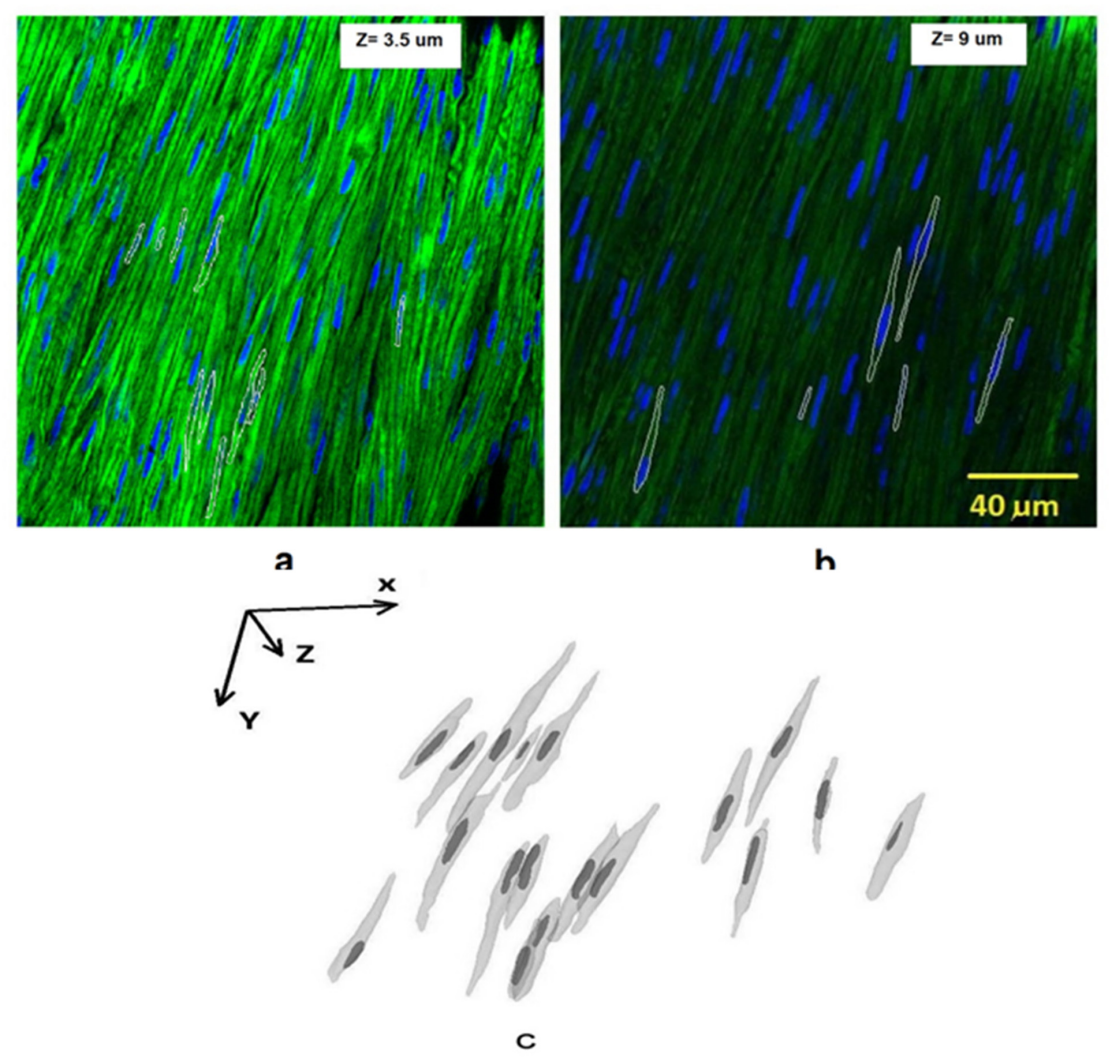
**a)** 3D cell contours in white were overlapped on original cell image. A slice at z = 3.5μm was analyzed. Blue indicates the nucleus, and green indicates the cell. **b)** A different slice at z = 9μm was analyzed to depict various layers of contours. **c)** 3D rendering of cells and nuclei from a representative sample. The XYZ Coordinate system was consistent with that in Fig 1. The viewpoint was taken from bottom to top along Z axis.

The original cell image and overlapped segmentation contours are shown in Fig 6a and 6b. Two slices were used to depict the contours of 3D object (the depths at z direction were 3.5 and 9 μm, respectively). The 3D structure of VSMCs is rendered in Fig 6c where the X, Y, Z coordinates align with that of the 2D image. The longitudinal and circumferential directions of vessel segment correspond to X and Y axis, respectively; when the radial direction corresponds to the Z direction in Fig 6c. Eighteen cells including the nuclei were reconstructed. Although some cells were very close to adjacent cells, they were distinguished by their respective nuclei (dark color). Since the largest contour of each cell was not always in the same plane, the contour sizes of cells were different.

**Fig 7:**
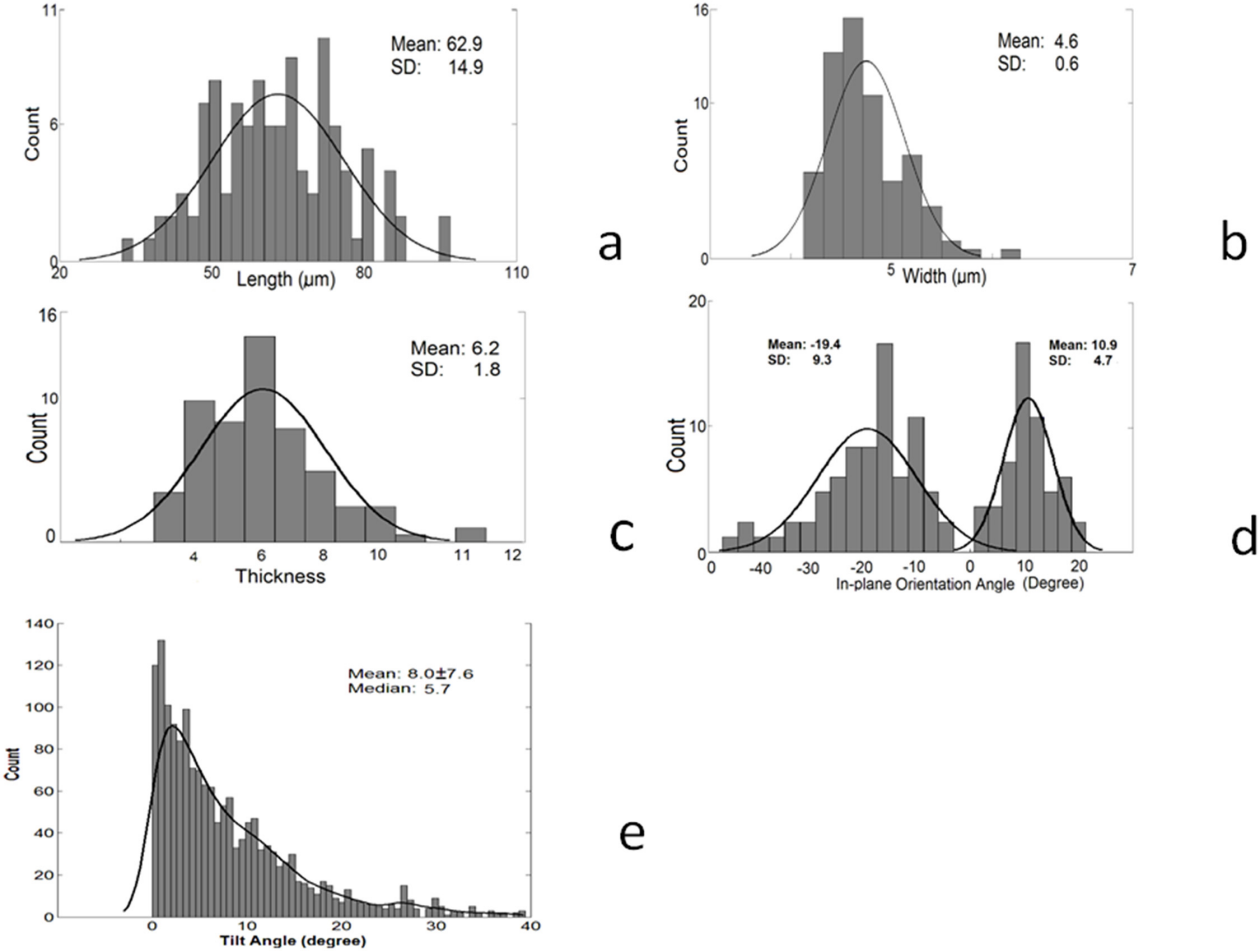
Various geometric parameters and orientation from cells: **a)** Length (μm). **b)** Width (μm); **c)** Thickness (μm), **d)** Orientation (degree) had two different values. **e)** Out-of-plane angle (degrees) histogram analyzed based on more than 1600 nuclei. Mean is the average value of measurement and SD is the standard variance. The normal distribution curve were fitted in the histograms in Fig 7a–7d, kernel distribution curve was fitted in Fig 7e.

The statistical distributions of morphometry of 112 VSMCs are shown in Fig 7a–7d for length, width, thickness, and orientation angle, respectively. The length, width and thickness (Fig 7a–7c) show approximately normal distributions with mean±SD of 62.9±14.9 μm, 4.6±0.6 μm and 6.2±1.8 μm, respectively, while the in-plane orientation distribution (Fig 7d) is a bimodal normal distribution (mixture of two normal distributions) with two different mean angles of -19.4±9.3° and 10.9±4.7°. The analysis of out-of-plane angles was based on more than 1,600 nuclei which shows a skewed distribution with a mean angle of 8.0±7.6° (median of 5.7°) (Fig 7e). Our results show that most VSMCs align off the circumferential direction with two nearly symmetric in-plane angels and with a small out-of-plane angle.

The geometrical parameters and orientation of VSMCs obtained by both the semi-automated method and manual measurements are summarized in Table 1. There was excellent agreement between segmentation algorithm and manual measurements. Measurements of orientation angle were nearly identical for both methods, while the difference between measurements of length and width were 2.7% and 4.7%, respectively. The comparison of intra-group measurements was summarized in Tables 2–4. If there was bimodal distribution of in-plane orientation angle in each heart, the comparison was listed in two columns in Table 3. Results show that the difference was < 10% for most parameters.

**Table 1:** Geometric data of vascular smooth muscle cells.

Number	Orientation (degrees)	Length (μm)	Width (μm)	Thickness (μm)	Slenderness Ratio
Algorithm (n = 112)	-19.4±9.3	62.9±14.9	4.6±0.6	6.2±1.8	12.3±4.3
	10.1±4.7				
Manual (n = 112)	-19.2±9.2	61.2±14.1	4.4±0.7		14.1±3.3
	11.1±4.3				

Orientation is the binary 2D region orientation projected from 3D data. Length and Width were computed on the 2D region. The values are summarized as average and standard deviation (mean±SD). The second row was the manual measurement result in 2D images.

**Table 2:** Geometric data measurement by each heart.

Heart Index	Cell Number	Length (μm)		Relative Difference(%)	Width (μm)		Relative Difference(%)
		Algorithm	Manual		Algorithm	Manual	
1	10	75.3	63.1	19.3	4.8	4.3	13.3
2	18	56.4	57.3	1.6	4.3	4.4	3.2
3	21	60.4	57.9	5.0	4.7	4.6	2.2
4	20	61.0	58.9	3.6	4.6	4.0	16.2
5	31	65.7	67.6	2.8	4.7	4.7	1.9
6	12	62.3	58.7	6.2	4.5	4.2	7.7

Difference Ratio was defined as: (Algorithm value minus Manual value) / Manual value.

**Table 3:** In-plane Orientation angle by each heart.

Heart Index	Cell Number	Orientation angle d1 (degree)		Relative Difference (%)	Orientation angle d2 (degrees)		Relative Difference (%)
		Algorithm	Manual		Algorithm	Manual	
1	10				8.7	9.0	2.8
2	18	-14.1	-14.9	5.3			
3	21	-16.4	-18.5	11.2	8.3	9.6	13.2
4	20	-18.0	-15.7	15.0	10.6	9.6	10.5
5	31	-19.4	-18.2	6.2	16.9	16.5	2.5
6	12	-29.8	-30.0	0.5			

**Table 4:** Out-plane Orientation angle by each heart.

Heart Index	Nuclei Number	Mean Tilt Angle (degree)	Standard Deviation
1	271	9.4	8.2
2	269	9.3	8.1
3	352	8.0	7.6
4	250	4.3	4.2
5	260	8.0	7.4
6	223	9.2	8.3

**Fig 8:**
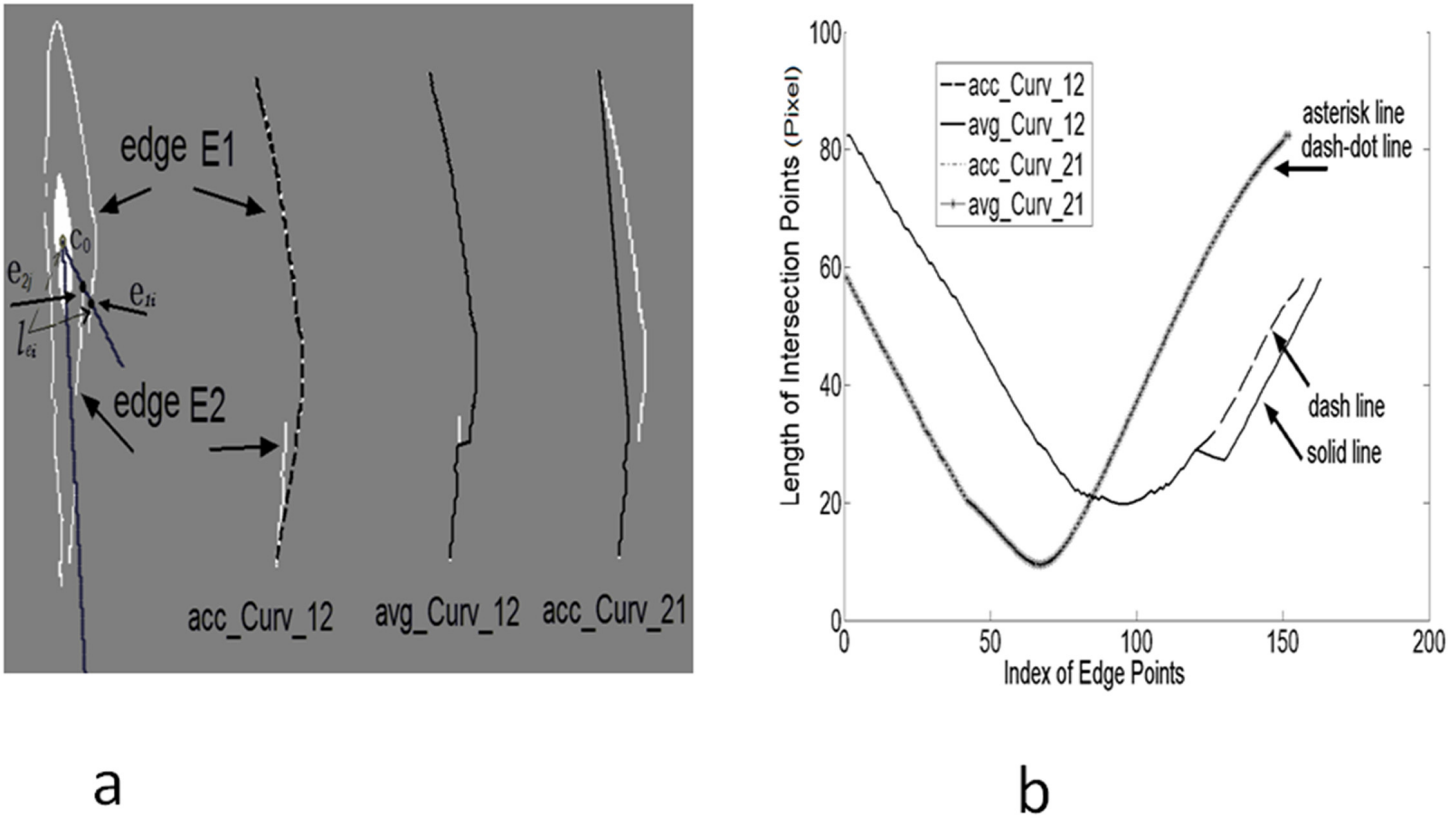
Four options of interpolation were compared: **a)** Interpolation results overlapped on cell image boundary in dark, unblocked e_2j_ and blocked points e_1i_ were drawn. Ray lines were dark, the longer ray line with 0°. l_ei_ was used to denote distance of intersection between point e_1i_ and center c_o_. **b)** Four options of interpolation were illustrated to show the intersection points length of interpolated edges.

For the interpolation procedure, we proposed a new algorithm of Bilateral Spline Interpolation (details are outlined in S1 Fig). An example is illustrated in Fig 8 to compare the measurement of accumulated curvature (acc_Curv) to average curvature (avg_Curv). The two circles (as noted by arrows e_2j_ and e_1i_) in the left cell of Fig 8a were unblocked and blocked intersection points, respectively. The three right columns in Fig 8a were the interpolated new edges (dark line), acc_Curv_12 means from edge 2 to edge 1. Different edge sequences and measurement methods were combined into all interpolation options. The lengths of intersection points were plotted in Fig 8b. Asterisk line (avg_Curv) overlapped with dash-dot line (acc_Curv) as their values were identical (Asterisk line and dash-dot line results were computed from avg_Curv_21 and acc_Curv_22 methods, respectively). The solid line (avg_Curv) and dash line (acc_Curv) had partial overlap. The dash line indicates the interpolated result is smooth; i.e., the connection position determined by acc_Curv may produce better result. For various directions, the final result was based on Standard Variance of intersection point lengths (SD). Hence, smaller standard variance and smaller curvature metric were obtained in the second column of Fig 8a. The measurement of acc_Curv, avg_Curv and SD from four interpolation options were {21.7, 0.1, 18.5}, {22.6, 0.1, 18.3}, {1.2, 0.0, 22.1}, {1.2, 0.0, 22.1}. The curvature computation unit was based on image pixel.

## Discussion

A 3D reconstruction of VSMCs is a significant challenge because of the complex cell geometries and orientation. Classical segmentation methods have been used in cross-sectional images [21–23] of blood vessel wall to reduce the risk of false recognition. These studies did not consider cellular structures, however, with orientation in the circumferential direction [24]. In present study, 112 cells were selected independently for the computer algorithm along with manual measurements for validations. The differences between the measurements of length and width were 2.7% and 4.7%, which confirmed the semi-automated algorithm is a reliable method for 3D cell extraction. The measurements of these 112 cells were also in line with previous measurements where additional 500 VSMCs were measured manually [19]. The differences between these measurements were 10.9% for length, 15.6% for width, and 19.5% for slenderness (propagation of error due to error in length and width). For orientation angles, the differences were 3.6% and 35.7% for the first and second peaks, respectively. The larger difference of the second peak was a result of nearly symmetric bimodal orientation distribution of 112 cells while the 500 cells followed a more symmetrical bimodal distribution. These statistical comparisons are reasonable given that the previous 500 cells were not the same as the 112 VSMCs used in the present study. For the measurement of tilt angle, the nucleus images were processed and then validated by the virtual phantom model. Over 1,600 nuclei were processed to verify statistical accuracy.

The 3D reconstruction of VSMCs showed that cells arrange with small out-of-plane angles (mean of 8°) in porcine coronary arteries, which is inconsistent with other studies [25]. Fujiwara and Uehara reported an average of 23° for the rat thoracic aorta and O'Connell et al. found a slightly lower value of 19° for the rat abdominal aorta [26]. These differences are not unexpected given that the former measurements were based on scanning electron microscopy (SEM) image of vessel cross-sections (2D) and that of the latter were based on 3D rendering of SEM images. Furthermore, the coronary arteries are muscular vessels while the aorta is an elastic vessel and hence there may be some intrinsic differences in VSMC arrangement. Our measurements revealed that VSMCs of porcine coronary arteries distribute largely on the longitudinal-circumferential plane following two nearly symmetric oblique orientation distributions.

The proposed segmentation algorithm addresses the limitations of 3D objects based on confocal microscopic image stacks. A 3D reconstruction of subcellular object was performed by Wood ST et al. [27], but they only reconstructed a single cell surrounded by background region. For a complex cell clump, however, automatic algorithms are only feasible under strict conditions. Therefore, image segmentation and importing of cell components [28] have been mostly completed by manual operation which is time-consuming and prone to human error. Here, we performed manual measurement to validate the proposed semi-automated method since the classical computer validation algorithms, like ratio cut [29], were not suitable. Most of validation algorithms are designed for natural images with closed contours. When used in VSMCs images, smaller and round cells were obtained and the longer tails may be missed. Generally, there are few morphology or geometry databases of muscle cell structure in literature or open cell databases, such as Murphy Lab-Data [30] or Madin-Darby Canine Kidney [31] to provide validation.

VSMCs have different shape patterns than cells processed in Voronoi-based methods [32–33]. VSMCs have fusiform shapes, which increase the overlap area of the cells. VSMCs on the longitudinal-circumferential plane prevent the seed point localization, such that methods of seed point selection, water shedding, or active contour for each cell cannot be employed [34]. Although Gestalt grouping [35] in computer vision is similar with VSMC edge linking, VSMCs have more complex features [36]. Edge grouping method has been mainly developed in non-biological image segmentation, and the cost function of edge closure is depended on a specific image. For example, some edge linking methods which search salient edges are not applicable to edges of cells [37–40]. The grouping effect relies on edge classification and proper connectivity graph model [41]. The edge based method is a good option (e.g., the edge blocking model) which localizes cell center as the basis for edge gap linking. There are also other similar model-based algorithms in edge or region detection, such as Active Segmentation with Fixation (ASF) [42] and the algorithms of Shen & Shi [43].

Many typical features of biological images are not applicable for extraction of VSMC characteristics. For instance, concave points are important to separate adjacent cells [44–48], but recognition of concave points is limited to stable and symmetric image features. For non-symmetric concave points, other shape information such as stable elliptical shape has been used to compose touching cells [47,49–50]. The proposed edge blocking model can combine position with shape information. Stable edges are used to restrict edges selecting in a local region and the accuracy can be improved by exploring more statistical features of 3D cell shape or spatial constraints. Although both edge and region information can improve the edge grouping robustness, region information cannot be used when cell intensity of neighboring cells are similar [37]. There are other segmentation strategies such as Ball Pivoting or construction of a convex hull from the detected edges and points, but model parameters are difficult to select. Therefore, 3D volume was obtained by traditional method of stacking 2D slice segmentation. Finally, mesh surface or 3D volume smoothing needs to be applied to overcome the inaccuracies of 2D segmentation. A reduction in the number of processed slices and the application of 3D interpolation on segmented slices can improve algorithm efficiency and should be investigated in the future.

In order to design more efficient image processing techniques, it is necessary to build a standard microscopy database of VSMCs by either manual or computerized methods as a reference. Group methods also require more machine learning algorithms [51]. Good classification of edges based on large data base can also help inform new feature recognition. Many advanced probability inference models cannot use the region patch or edge feature in VSMC images due to lack of validation data or training data sets.

## Limitations of Study

The detailed analysis of inter- vs intra-group variability was only based on limited number of cells in some samples in all 6 hearts. It is not sufficient for a rigorous statistical analysis. Based on imaging quality limitations, the number of segmented cells in some samples was small. This is a limitation that will need to be addressed with collection of greater sets of data. The vascular smooth muscles can alter their geometry with contraction and tissue fixation can also distort the geometry. Here, we ensured that the VSMC were fully relaxed and the tissues were not physically clamped. Finally, since there is no gold standard for 3D reconstruction of VSMCs, we used a manual method for validation of the 3D algorithm. A synthetic data was used to specifically validate the measurement of out-of-plane angles that was not amenable to the 2D manual process.

## Summary

The VSMC morphology was extracted from confocal microscopy image stacks by using a 3D segmentation algorithm. Cell segmentation was decomposed into several procedures, including block detection and edge growing. The edge classification was based on shape and position relation. Semi-automation method was proposed to overcome the failure of edge detection, where the region of interest was applied and the user can select key edges for edge growing. The 3D reconstruction was validated by manual measurement. The 3D database of VSMC geometry can be utilized for better understanding of the physiology and biomechanics of coronary arteries.

Matlab scripts of the core algorithm were uploaded to public website: http://dx.doi.org/10.6084/m9.figshare.1504123.

## Appendix

### Edge Processing

For non-uniform intensity problem resulting from fluorescent indicator density drop off, 2D deconvolution tool was used in some overly-bright slices where 3D deconvolution may miss subtle edges and cause ring artifacts. Coherent filtering was then used for local region smoothing and edge was extracted by Canny detector.

Other preprocessing included bifurcation removal, spur removal and single-pixel edge creation such that an edge can only have two end points. The order of edges was the same as the ray angle in a clock-wise direction. Edges were classified in one of three categories: 1) Type A: Real edge with positive or negative second order derivative; gradient was pointed to cell center; disconnected; 2) Type B: False edge from other cell, with incorrect second order derivative; nearby real edge was not detected by Canny as obscure feature; and 3) Type C: Touching edge with other cell, but with same derivative as current cell. Positive or negative second order derivatives were denoted at left or right side of cell, respectively; e.g., right side edge’s gradient direction must point to left, where cell region is located with higher intensity. The line fitting of high order derivative extreme points in edge were used an alternative technique for Canny detector to find real cell edge. For instance, Laplace operation was used along specific profile to search local extreme points. The details are provided in the next section.

### Edge Growing

Edge growing algorithm is the keystone of segmentation where the growing was defined as a new line or curve which connected one edge’s top with another edge’s bottom. According to edge and gap categories, major techniques were based on interpolation and PCA analysis. Canny edge from other cell was sometimes incorrectly identified to belong to current cell edge. The white circle in S1A Fig denotes the complex edge patterns located at the top of a nucleus. Edge indicated by green was from current cell and was from another cell (Type B edge). The real edge point was indicated by red, and it was detected by second-order derivative not by Canny. The yellow indicated edge detected by both Canny detector and Laplace. In fact, yellow was Type A edge which was at the right trunk or tail edge in this figure.

#### 1) Laplace Verification PCA

To restore new edge at red point, Laplace Verification PCA method was proposed as follows:

1) Select points from sampling region surrounding by consecutive ray lines (within 5–10°).
2) *Original image intensity profile along ray $R_{c_{0}e_{i}}$ was processed by Laplace* Δ.
3) *Coordinate p*_*i*_ *with value I*_*pi*_ ≥ 0(*or I*_*pi*_ *≤* 0)*was selected as below* Eq (A1).
4) *Select initial sampling window (5× 5 window) centered at one position p*_*i*_ *where all positions were analyzed by Principal component analysis on covariance matrix (PCACOV)*.
5) *Principal component vector v*_1_ *was set equal to the direction of verified edge in the local window as in* Eq (A2). *New edge was drawn by setting its start point at geometry center of all p*_*i*_ *in the local window*. *The direction was selected as the principal component vector and the edge length was the maximal distance between center and to all p*_*i*_.
6) *After new edge was drawn*, *local sampling window was translated to the end point of new edge for next sampling*, *go back to step 1)*.
7) *The loop was terminated after ray lines were processed or gap was filled*.

$$\left\{I_{p_{i}}|I_{p_{i}}\in I_{p},\;I_{p}=\Delta \left(R_{c_{0}e_{i}}\right),I_{p_{i}}\geq 0\;or\;I_{p_{i}}\leq 0\right\}$$ (A1)

$$\left|v_{1},v_{2}\right|=pcacov\left(p_{i}\right)$$ (A2)

*p*_*i*_ was restricted between nucleus edge and Type B edge along profile line.

#### 2) Bilateral Spline Interpolation

The type I gap was filled by Bilateral Spline Interpolation. Spline interpolation [52] was computed between the unblocked edges pair (*E*_1_, *E*_2_), *E*_1_ = {*e*_11_,…,*e*_1*m*_}, *E*_2_ = {*e*_21_,…,*e*_2*n*_}. Gap was between (*e*_1*m*_, *e*_21_) and parameter tension was set to zero. The 2D position *p*_*e*_ was element of interpolation as in Eq A3.

$$\{p|p_{1}\cup p_{2}\},\;p_{1}=\left\{p_{e_{11}},\ldots ,p_{e_{1i}},\ldots ,p_{e_{1m}}\right\},\;p_{2}=\left\{p_{e_{21}},\ldots ,p_{e_{2j}},\ldots ,p_{e_{2n}}\right\}$$ (A3)

Bilateral means twice interpolation; i.e., from p1 to p2from p2 to p1, all elements in *p*_*1*_ were selected, but in *p*_*2*_, elements were from *e*_2*j*_ to the end of *e*_2*n*_, *e*_2*j*_ was not fixed at one edge point. Measurement of curvature *κ* of all interpolation results was obtained by moving a start point to a different position *e*_2*j*_. It was depicted as follows:

$$argmin_{j}\sum \left(\kappa_{e}^{2}\right),\;\{e|e_{11}\leq e\leq e_{1m},\;e_{1m}<e_{2j},\;e_{2j}\leq e\leq e_{2n}\}$$ (A4)

The operator ≤ means the order of points is as follows: first ≤ second, second ≥ first. The accumulated curvature of edge point and the length-normalized curvature were sought to determine the edge with the minimum value. For interpolation *from p*_*2*_ *to p*_*1*_, the measurement was obtained as below:

$$argmin_{i}\sum \left(\kappa_{e^{\prime }}^{2}\right),\;\{e^{\prime }|e_{2n}\geq e^{\prime }\geq e_{21},\;e_{21}<e_{1i},\;e_{1i}\leq e^{\prime }\leq e_{11}\}$$ (A5)

New linked edges *e* and *e*′ in Eqs (A4) and (A5) were converted into *l*_*e*_ and *l*_*e′*_, where the value *l*_*e*_ was the length between intersection point and center. The final selection was based on smaller standard variance of *l*_*e*_ and *l*_*e′*_. The illustrated procedure was referred in Fig 7.

#### 3) Alternative method

For tail edge, another Splitting approach was employed. An example of Type C edge was depicted in S1C Fig. The edges of two adjacent cells merged into a single edge. After connecting the edge and three auxiliary lines (thick red lines), they formed a closed polygon ABCDEF. The shortest distance from edge to line BD (i.e.; CF in S1D Fig) were determined by either area or length of line segment. The original edge EFA was split at F and treated as a new edge ready for edge linking.

### Stable Edge

The stable edge algorithm identified the same edge in different slices. If two edges in two slices belonged to the same cell, one edge was taken as seed, while another edge in the second slice could be sought as stable edge in the same position or within a local window. Although edges may move slightly, there were no abrupt position changes along the slices. The linking of stable edges was based on same method mentioned above. When there was no stable edge found, an alternative method (e.g., interpolation, PCA analysis, etc.) was used to find the edge (as described in above section).

The searching neighbor was usually set as 3 or 5 pixels square. Computation overlap of point was defined in Eq (A6) and the stability of point was given as Eq (A7):

$$O\left(e_{i},e_{0}\right)=1-\frac{min\left(d\left(e_{i},e_{0}\right)\right)}{w},\;e_{0}\in E_{0}$$ (A6)

$$S=\frac{1}{M}\sum_{j=1}^{M}O\left(e_{i},e_{0}\right)$$ (A7)

*w* is the neighbor window width, *d*(*e*_*i*_, *e*_0_) is distance from point *e*_*i*_ to any point *e*_*0*_ in seed edge. *M* was the number of slices. Point with maximal *0*(*e*_*i*_, *e*_0_) along one ray line was taken as a stable point. An edge was regarded as stable edge when the ratio of its stable points was more than 30% or other threshold. Below this threshold, edge was regarded as an unstable noise[1].

### Validation of Tilt Angle Measurement

The virtual phantom, an idealized ellipsoid, was considered. The ellipsoid is determined by a group of 2D curves with particular parameters, including semi-major axis and semi-minor axis. Generally, it can be rotated to an arbitrary angle by matrix operation and deformation on any sites. In our explanatory model, the semi-major axis, semi-minor axis and rotation angle of the ellipsoid were chosen within the range of cellular parameters (as shown in Fig 7). Moreover, mesh deformation was considered on the ellipsoid surface to simulate the irregular part of the boundary. The tilt angle of an ellipsoid is identical to the vector angle of semi-major axis. The virtual phantom was modified based on an open-source code in MATLAB file exchange. The program of the validation model was uploaded to the same website in figshare.

The surface of ellipsoid was decomposed into 3D voxel in sequential slices and 2D images were generated. Using the proposed segmentation algorithm, a 3D object was reconstructed based on obtained 2D images. The process of decomposing mesh surface into voxel was called Voxelization. A tilt angle was measured based on 3D voxel and compared with the predefined rotation angle of the ellipsoid. The PCACOV-based measurement method was therefore validated.

The reconstructed geometry based on 3D voxel is not as smooth as the ellipsoid mesh since high curvature was not preserved in Voxelization. Although the induced tilt angle of 3D voxel departed from a predefined angle, the deviation was very slight. S1E–S1H Fig shows the difference between the predefined and 3D voxel-measured tilt angles to be less than 10%. When considering size normalization, PCANormCOV lead to a less accurate result than that of PCACOV. Moreover, the dimension of the ellipsoid has little influence on the measurements. There was no difference found between the ellipsoid with two different long axes: 70 and 40 pixel, respectively.

Although a real nucleus is not an idealized ellipsoid and is irregularly shaped, the measurement is analogous to that of an ellipsoid since the measurement is largely determined by the definition of tilt angle. For an ellipsoid composed of cross-sectional rings along the long semi-axis direction and has an orientation angle for each ring center, the overall tilt angle is the average over all rings. A deformed ellipsoid was tested to simulate irregular nucleus geometry. One end of the ellipsoid was rotated by deformation angle around X axis and the other end was fixed, as the ellipsoid was then deformed using Laplacian interpolation [53]. With the increase of deformation angle, it showed that there was no difference between the change of tilt angles of deformed ellipsoid and the corresponding voxel data.

## Supporting Information

S1 Fig **A.** Edge map for original cell image, Canny edge was green, Laplace operation result was red point, yellow was the overlapped edge which denoted correct edge in boundary. **B.** Original image, cell was green and nucleus was blue, white circle showed the compared regions of current cell. **C.** Original image with arrow indicated skeleton. **D.** AFE was edge points from two cells and will be split. ABCDEF were the auxiliary points to test the shortest distance from AFE to BCD, F was at the shortest position. AB and ED were vertical to its own cell vector. Blue indicates the nucleus, and green indicates the cell. **E.** Ellipsoid mesh surface as digital phantom. **F.** 3D Render of Voxel object after voxelization of Ellipsoid. **G.** Rotated Ellipsoid with a tilt angle. **H.** Difference between measured and predefined tilt angle. Two different measurement methods were used. PCANormCOV 70 indicated size normalization and PCACOV 70 indicated non-normalization, 70 was length along Y axis.
(TIF)
