# Supplementary figures and images for: 3D Reconstruction of Coronary Artery Vascular Smooth Muscle Cells
Source: PLoS One. 2016 Feb 16;11(2):e0147272. doi: 10.1371/journal.pone.0147272 (PMC4755581; doi:10.1371/journal.pone.0147272)

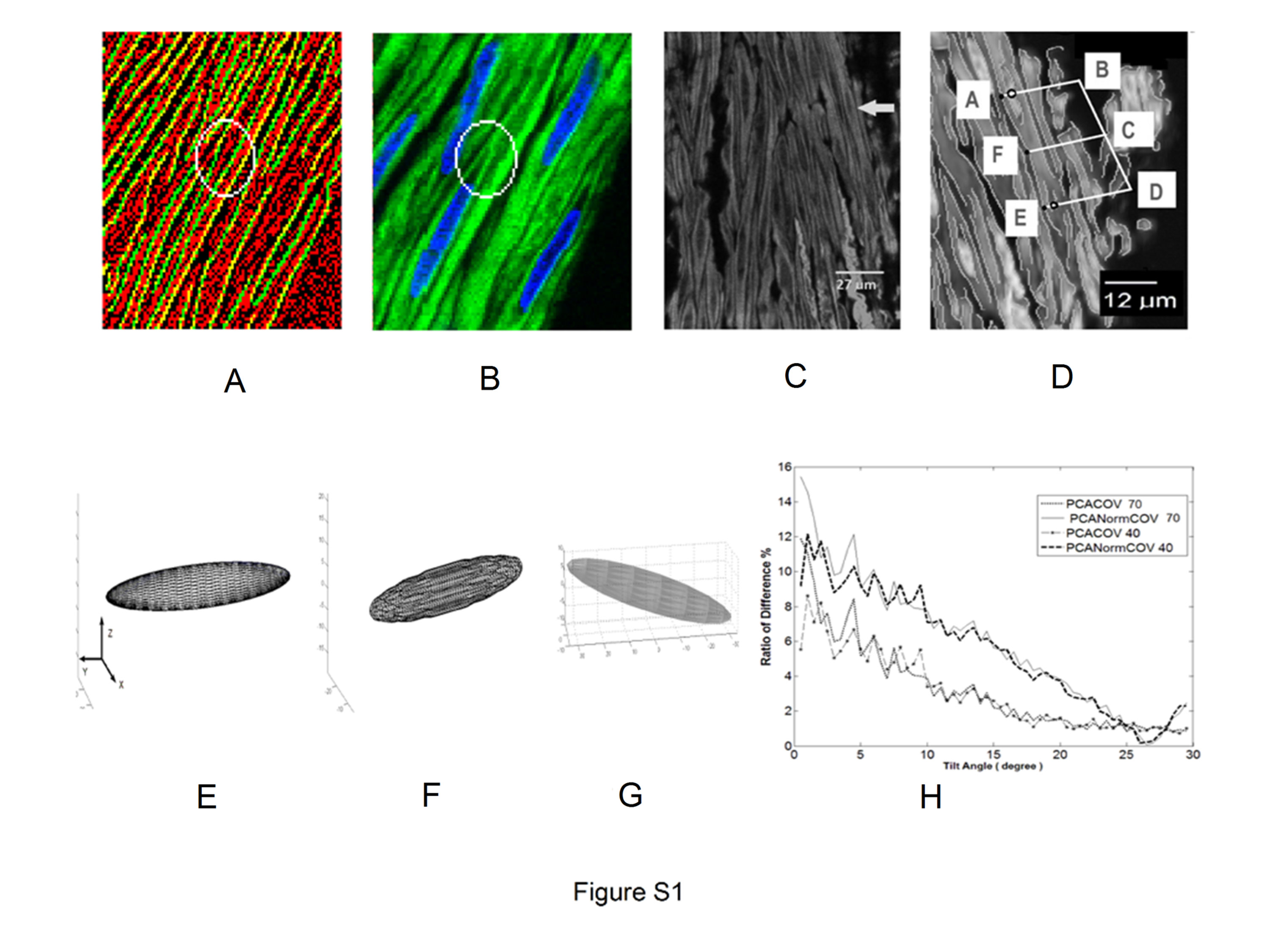

Supplement: S1 Fig — A. Edge map for original cell image, Canny edge was green, Laplace operation result was red point, yellow was the overlapped edge which denoted correct edge in boundary. B. Original image, cell was green and nucleus was blue, white circle showed the compared regions of current cell. C. Original image with arrow indicated skeleton. D. AFE was edge points from two cells and will be split. ABCDEF were the auxiliary points to test the shortest distance from AFE to BCD, F was at the shortest position. AB and ED were vertical to its own cell vector. Blue indicates the nucleus, and green indicates the cell. E. Ellipsoid mesh surface as digital phantom. F. 3D Render of Voxel object after voxelization of Ellipsoid. G. Rotated Ellipsoid with a tilt angle. H. Difference between measured and predefined tilt angle. Two different measurement methods were used. PCANormCOV 70 indicated size normalization and PCACOV 70 indicated non-normalization, 70 was length along Y axis. (TIF) [file pone.0147272.s001.TIF]
